# Association of systemic inflammation with major adverse cardiovascular events in patients with acute myocardial infarction

**DOI:** 10.1016/j.ajpc.2026.101574

**Published:** 2026-03-24

**Authors:** Chi Nguyen, Amanda M. Ackermann, Erica Marieb, Jeffrey R. Skaar, Wing Chow, Radha Ryali, Lyuba Popadic, Xiyuan Wu, Xinshuo Ma, Kathleen E․ Kearney

**Affiliations:** a Novo Nordisk Inc, Plainsboro, NJ, USA; b Komodo Health, New York, NY, USA; c University of Washington School of Medicine, Seattle, WA, USA

**Keywords:** Systemic inflammation, Acute myocardial infarction, Cardiovascular disease, Real-world evidence, Cohort study, Adults

## Abstract

**Objective:**

Assess the association of systemic inflammation (SI) with major adverse cardiovascular events (MACE) and heart failure (HF) in patients with acute myocardial infarction (AMI).

**Methods:**

This retrospective cohort study included adults hospitalized for type 1 AMI from July 2016 through December 2023 in the Komodo Healthcare Map database. High-sensitivity C-reactive protein (hsCRP) test results 1 year before or after admission determined SI status (with SI, ≥1 test 2–10 mg/L; without SI, all tests <2 mg/L). hsCRP tests within 30 days post-AMI or hospitalization, associated with antimicrobial or corticosteroid use, or with values >10 mg/L were excluded. Incidence of revised 3-point MACE (nonfatal MI, nonfatal stroke, or all-cause mortality), revised 4-point MACE (nonfatal MI, nonfatal stroke, urgent revascularization, or all-cause mortality), and HF were estimated per 1000 person-years (PY). Cox regression models assessed the adjusted association between SI and the outcomes.

**Results:**

Among 3149 eligible patients (mean age, 61 years), 46.4% had SI. Compared with patients without SI, patients with SI were more likely to be female and have more comorbidities. Patients with SI had a higher incidence of revised 3-point (45.4 vs. 27.3/1,000PY) and 4-point MACE (52.1 vs. 35.1/1,000PY). SI was associated with increased risk of revised 3-point and 4-point MACE (HR=1.39 [95% CI, 1.08–1.78] and 1.26 [1.01–1.58], respectively). Among patients without prior HF (*n* = 1988), SI was associated with increased risk of HF hospitalization (HR=2.04 [1.26–3.30]).

**Conclusions:**

Patients with AMI and SI have an increased risk of MACE and incident HF hospitalization compared with patients with AMI without SI.

## Graphical abstract

**Figure:**
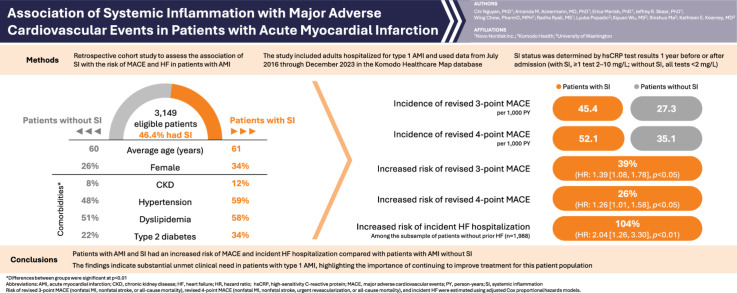

## 1 Background and Methods

### 1.1 Introduction

Acute myocardial infarction (AMI) is a leading cause of morbidity and mortality in the United States. >800,000 AMIs occur annually, of which 200,000 are recurrent [1]. While advances in treatment have improved AMI survival, substantial morbidity and mortality remain, with the potential for recurrent major adverse cardiovascular events (MACE), leading to hospitalization or death [[2], [3], [4], [5]]. Among patients who have experienced an AMI, 5%–9% will experience a subsequent AMI, and 14% will die within 1 year; the 5-year risk of subsequent MACE (nonfatal MI, nonfatal stroke, and CV-related death) is 33.4% [3,6]. Due to structural and functional damage that occurs after infarction, patients with AMI are also at a higher risk of developing heart failure (HF). Research indicates that 20%–30% of patients will develop HF within a year [7,8]. The frequency of MACE and HF after AMI underscores the need to identify modifiable risk factors that contribute to these outcomes.

Systemic inflammation (SI) plays a role in the initiation and progression of atherosclerotic plaques and is a risk factor that may be associated with AMI and subsequent cardiovascular events [9,10]. In patients who have experienced an AMI, the acute inflammatory response is a critical component of myocardial healing as it facilitates the clearance of necrotic tissue and initiates cardiac remodeling [11]. After the acute phase following an AMI, the inflammatory response typically declines gradually and stabilizes within 2–4 weeks [12]. However, some patients have chronically elevated SI, which has been implicated in pathogenic post-infarction tissue remodeling (e.g., infarct expansion, myocardial thinning, left-ventricular dilatation, systolic or diastolic dysfunction) and may contribute to subsequent cardiovascular events [[13], [14], [15], [16], [17]]. In the clinical setting, SI is typically identified by C-reactive protein (CRP), which is produced by the liver in response to circulating pro-inflammatory cytokines, especially interleukin-6 (IL-6) [18]. For the assessment of cardiovascular inflammation, CRP is usually measured in the clinical setting by high-sensitivity CRP (hsCRP) testing [[19], [20], [21]]. Elevated IL-6 levels are associated with increased risk of cardiovascular events, cardiovascular mortality, and all-cause mortality in patients with acute coronary syndrome [22,23]. Elevated hsCRP levels have also been shown to predict the occurrence of MACE in patients with atherosclerotic cardiovascular disease (ASCVD), independent of low-density lipoprotein cholesterol (LDL-C) [[24], [25], [26]]. Therefore, the association between systemic inflammation and cardiovascular outcomes among patients with AMI may be important for clinical decision-making and treatment planning to reduce the risk of these events.

Cardiovascular inflammation is recognized as a therapeutic target for reducing the risk of future events in at-risk patients [27]. The CANTOS (Canakinumab Anti-inflammatory Thrombosis Outcome Study), COLCOT (Colchicine Cardiovascular Outcomes Trial), and LoDoCo2 (Low-dose Colchicine 2) trials demonstrated that anti-inflammatory therapies can effectively reduce the risk of cardiac events among at-risk patients [[28], [29], [30]]. However, the prevalence and association of SI with recurrent cardiovascular events have been primarily studied in the context of randomized trials and have not been well characterized in broader populations. Therefore, there is a need for large, population-based epidemiological studies to clarify the association between SI and subsequent cardiovascular events in patients who have experienced an AMI. Such studies are particularly relevant concerning type 1 AMI, a major contributor to overall AMI incidence and for which SI may be pivotal, given its involvement in the rupture of atherosclerotic plaques.

The primary objective of this study was to assess the association between SI, as indicated by hsCRP levels, and the risk of MACE among patients with type 1 AMI. The secondary objective was to assess the association between SI and the risk of incident HF among patients with type 1 AMI without a history of HF.

### 1.2 Methods

This retrospective, longitudinal, observational study used the Komodo Healthcare Map [31], which includes deidentified administrative medical and pharmacy claims linked with clinical and laboratory measurements for a nationally representative US population, from January 1, 2016, through December 31, 2023. Mortality data are sourced from multiple databases, including the Death Master File provided by the Social Security Administration, inpatient discharge status, and third-party obituary data. This study used deidentified data and was exempted from institutional review board review. The research team accessed a limited dataset without patient-identifiable information, and only summary statistics were reported. This work was conducted in accordance with the Declaration of Helsinki and complied with all relevant provisions of the US Health Insurance Portability and Accountability Act (HIPAA). Komodo Health approved the use of the dataset for this study.

This study included patients with ≥1 inpatient admission with an International Classification of Diseases, Tenth Revision, Clinical Modification (ICD-10-CM) diagnosis code for type 1 AMI in the primary position (I21.0, I21.1, I21.2, I21.3, I21.4, or I21.9). ICD-10-CM codes were also used to further identify patients by AMI subtype, including ST-segment elevation myocardial infarction (STEMI; codes I21.0, I21.01, I21.21, I21.29, I21.09, 410.81, I21.11, I21.19, I21.3, I21.02, I21.1, or I21.2 in the primary position) and non–ST-segment elevation myocardial infarction (NSTEMI; code I21.4 in the primary position). The index date was the first occurrence of AMI during the patient identification period (July 1, 2016, to December 31, 2023). Eligible patients were aged ≥18 years on the index date and had continuous health plan enrollment ≥6 months before and ≥1 day after the index date (**Supplemental Figure 1**). SI status was determined based on hsCRP results during 1 year before or after the AMI index date (without SI: all hsCRP tests <2 mg/L; with SI: ≥1 hsCRP test 2–10 mg/L) [28,32]. Prior studies show that single elevated hsCRP tests are associated with cardiovascular outcomes; based on this literature, a single eligible hsCRP value was considered for classification of SI in this study [33,34]. hsCRP tests >10 mg/L or taken within 30 days after an AMI event, inpatient visit, or emergency department (ED) visit were excluded due to potential association with acute inflammation [24,35,36]. Additionally, hsCRP tests were excluded if there was evidence of antibiotic, antiviral, or antimycotic medication use within 2 weeks before or 1 week after the test date or evidence of corticosteroid use within 1 month before the test date. Patients were excluded if they had coronary artery bypass grafting during the index hospitalization or any of the following conditions during 1 year before or after the index date: end-stage renal disease, severe hepatic disease (hepatic encephalopathy, ascites, or hepatic cirrhosis), evidence of chronic infectious disease (hepatitis, HIV, tuberculosis), or cancer (except skin cancer). For the incidence HF analysis, patients with prior HF were excluded based on diagnosis codes for HF and HF-related conditions, such as rheumatic heart disease, hypertensive heart disease with heart failure, hypertensive heart and chronic kidney disease with heart failure, and cardiomyopathy.

Patient demographic and clinical characteristics, including relevant comorbidities, procedures, and medications, were evaluated during the baseline period (6 months preindex to the index date). Demographic characteristics included age in years, sex, race, ethnicity, insurance type (commercial, Medicaid, Medicare), and geographic region (Northeast, Midwest, South, West, Other). Clinical characteristics included the Quan-Charlson Comorbidity Index (QCI) [37]; comorbidities related to AMI (e.g., hypertension, dyslipidemia, type 2 diabetes, obesity, prior MI, chronic obstructive pulmonary disease, peripheral vascular disease, chronic kidney disease [CKD], and autoimmune diseases) were identified from medical claims using ICD-10-CM codes. Medication use, ascertained from pharmacy claims using the national drug codes, included cardiovascular medications, immunosuppressive medications, and nonsteroidal anti-inflammatory drugs (see **Supplemental Table 1** for a list of medications).

The primary study outcomes were the occurrence of revised 3-point MACE (nonfatal MI hospitalization, nonfatal stroke hospitalization, or all-cause mortality) and revised 4-point MACE (nonfatal MI hospitalization, nonfatal stroke hospitalization, urgent revascularization, or all-cause mortality). Revascularization was identified using ICD-10 procedure codes (ICD-10-PCS), current procedural terminology (CPT), or Healthcare Common Procedure System (HCPCS) for percutaneous coronary intervention or coronary artery bypass grafting. Urgent or unplanned procedures were defined as claims originating in the emergency department or emergency department that later admitted to hospital.

Subgroup analyses were conducted by AMI subtype (STEMI and NSTEMI). Additionally, individual MACE components (nonfatal MI, nonfatal stroke, all-cause mortality, and urgent revascularization) were reported. The secondary study outcome, assessed only among patients without HF prior to or during the index AMI hospitalization, included assessment of incident HF, defined as either hospitalization or outpatient visit during follow-up with an ICD-10-CM diagnosis code for HF (I50x) in any position.

Descriptive statistics (mean and standard deviation for continuous variables and counts and percentages for categorical variables) were reported for patient demographic and clinical characteristics at baseline. Incidence rates were calculated as the number of patients who experienced a new event during follow-up divided by the total time at risk for all patients. Incidence rates with 95% confidence intervals (CI) were reported per 1000 person-years (PY). For each outcome, hazard ratios (HRs) comparing patients with SI versus patients without SI (reference group) were estimated using Cox proportional hazards models, adjusting for baseline covariates, including patient demographic characteristics, comorbidities, QCI, and medication use. HRs were reported with corresponding 95% CIs. In the subgroup analysis of STEMI versus NSTEMI, we used Cox proportional hazards models with an interaction term (SI × subgroup) to test for effect modification and calculate hazard ratios for each subgroup. A p-value <0.05 was considered statistically significant. Analyses were conducted in R 4.3.3 with the “survival” package 3.7–0.

## 2 Results

### 2.1 Cohort baseline characteristics

Among 3149 eligible patients, 46.4% (*n* = 1461) had SI (Table 1; **Supplemental Table 2; Supplemental Figure 1**). Of the 3149 patients, most patients (60.8%) had hsCRP measured after an AMI, 31.5% had hsCRP measured before an AMI, and 242 patients (7.7%) had eligible hsCRP tests both before and after an AMI . In the overall sample, the median (interquartile range) hsCRP was 3.40 (2.30, 5.60) mg/L in the SI group and 0.80 (0.40, 1.20) mg/L in the non-SI group. Patients with and without SI were similar in age (mean age: 61 vs. 60 years, respectively). Compared with patients without SI, patients with SI were more likely to be female (34% vs. 26%) and to have more comorbidities, including CKD (12% vs. 8%), hypertension (59% vs. 48%), dyslipidemia (58% vs. 51%), and type 2 diabetes (34% vs. 22%) (all *p* < 0.01). There was no significant difference in autoimmune disease between the two cohorts (4.4% vs. 3.8%; *p* = 0.4) (Table 1), but patients with SI were more likely to use immunosuppressive medication (25% vs. 20%; *p* < 0.01). Patients with SI were also more likely to use cardiovascular medications, including antihypertensives (60% vs. 51%; *p* < 0.01), antiplatelets (15% vs. 11%; *p* < 0.01), and anticoagulants (9.4% vs. 7.5%; *p* = 0.045). The average follow-up time between patients with and without SI was similar (31 vs. 32 months, respectively).

**Table 1:** Cohort demographic and clinical characteristics at baseline, overall. and by systemic inflammation status.

	Patients with AMI^1^^,^^2^				Patients with AMI without prior HF			
Characteristic	All eligible patients with AMI (*N* = 3149)	AMI without SI (*n* = 1688)	AMI with SI (*n* = 1461)	p-value	All eligible patients with AMI (*n* = 1988)	AMI without SI (*n* = 1120)	AMI with SI (*n* = 868)	p-value
**Age, mean (SD)**	61 (11)	60 (11)	61 (11)	0.072	59 (11)	59 (10)	59 (11)	>0.9
**Age group**^3^**, n (%)**				0.16425				0.5
18–44	223 (7.1)	121 (7.2)	102 (7.0)		162 (8.1)	91 (8.1)	71 (8.2)	
45–54	634 (20.1)	339 (20.1)	295 (20.1)		435 (21.8)	236 (21.0)	199 (22.9)	
55–64	1302 (41.3)	726 (43.0)	576 (39.4)		879 (44.2)	513 (45.8)	366 (42.1)	
65–74	588 (18.7)	305 (18.1)	283 (19.4)		332 (16.7)	182 (16.2)	150 (17.3)	
75+	402 (12.8)	197 (11.7)	205 (14.0)		180 (9.1)	98 (8.8)	82 (9.4)	
**Gender, n (%)**				<0.001				0.002
Male	2184 (69.4)	1231 (72.9)	953 (65.2)		1412 (71.0)	829 (74.0)	583 (67.2)	
Female	932 (29.6)	438 (25.9)	494 (33.8)		560 (28.1)	281 (25.0)	279 (32.1)	
Unknown/missing	33 (1.0)	19 (1.1)	14 (1.0)		16 (0.8)	10 (0.9)	6 (0.7)	
**Race, n (%)**				0.006				0.033
White/Caucasian	1879 (59.6)	983 (58.2)	896 (61.3)		1175 (59.1)	651 (58.1)	524 (60.4)	
Black/African American	242 (7.7)	113 (6.7)	129 (8.8)		128 (6.4)	59 (5.3)	69 (7.9)	
American Indian, Alaska Native, or Asian	197 (6.3)	121 (7.2)	76 (5.2)		123 (6.2)	78 (7.0)	45 (5.2)	
Other	245 (7.8)	134 (7.9)	111 (7.6)		163 (8.2)	93 (8.3)	70 (8.1)	
Unknown/missing	586 (18.6)	337 (20.0)	249 (17.0)		399 (20.1)	239 (21.3)	160 (18.4)	
**Ethnicity, n (%)**				<0.001				0.006
Hispanic	410 (13.0)	195 (11.6)	215 (14.7)		242 (12.1)	118 (10.5)	124 (14.3)	
Non-Hispanic	2195 (69.7)	1158 (68.6)	1037 (70.9)		1389 (69.8)	781 (69.7)	608 (70.0)	
Unknown/missing	544 (17.3)	335 (19.8)	209 (14.3)		357 (17.9)	221 (19.7)	136 (15.7)	
**Insurance type, n (%)**				<0.001				0.016
Commercial health insurance	1948 (61.9)	1122 (66.4)	826 (56.5)		1370 (68.9)	800 (71.4)	570 (65.7)	
Medicare (Advantage and FFS)	871 (27.7)	418 (24.7)	453 (31.0)		429 (21.5)	227 (20.2)	202 (23.3)	
Medicaid	330 (10.5)	148 (8.8)	182 (12.4)		189 (9.5)	93 (8.3)	96 (11.1)	
**Geographic region, n (%)**				0.02				0.4
Northeast	687 (21.8)	334 (19.7)	353 (24.1)		428 (21.5)	231 (20.6)	197 (22.7)	
Midwest	379 (12.0)	210 (12.4)	169 (11.5)		241 (12.1)	144 (12.8)	97 (11.2)	
South	1244 (39.5)	672 (39.8)	572 (39.1)		793 (39.8)	441 (39.3)	352 (40.6)	
West	839 (26.6)	472 (27.9)	367 (25.1)		526 (26.4)	304 (27.1)	222 (25.6)	
**Quan-Charlson Comorbidity Index (QCI)**								
Mean (SD)	0.62 (1.13)	0.51 (1.02)	0.75 (1.24)	<0.001	0.32 (0.69)	0.28 (0.66)	0.38 (0.73)	<0.001
0, n (%)	2143 (68.1)	1238 (73.3)	905 (61.9)	<0.001	1544 (77.6)	904 (80.7)	640 (73.7)	0.003
1, n (%)	469 (14.9)	203 (12.0)	266 (18.2)		293 (14.7)	140 (12.5)	153 (17.6)	
2, n (%)	308 (9.8)	151 (8.9)	157 (10.7)		116 (5.8)	59 (5.3)	57 (6.6)	
3+, n (%)	229 (7.3)	96 (5.7)	133 (9.1)		35 (1.8)	17 (1.5)	18 (2.1)	
**Other comorbid conditions,**^4^**n (%)**								
CKD	299 (9.5)	129 (7.6)	170 (11.6)	<0.001	118 (5.9)	62 (5.5)	56 (6.5)	0.4
Dyslipidemia	1701 (54.0)	858 (50.8)	843 (57.7)	<0.001	1003 (50.4)	538 (48.0)	465 (53.6)	0.014
Hypertension	1686 (53.5)	818 (48.5)	868 (59.4)	<0.001	963 (48.4)	502 (44.8)	461 (53.1)	<0.001
Heart failure	205 (6.5)	89 (5.3)	116 (7.9)	0.002				
Atrial fibrillation	183 (5.8)	95 (5.6)	88 (6.0)	0.6	66 (3.3)	36 (3.2)	30 (3.5)	0.8
Selected autoimmune disease^5^	128 (4.1)	64 (3.8)	64 (4.4)	0.4	80 (4.0)	41 (3.7)	39 (4.5)	0.3
Obesity	647 (20.5)	256 (15.1)	391 (26.7)	<0.001	358 (18.0)	144 (12.9)	214 (24.7)	<0.001
Smoking	428 (13.6)	193 (11.4)	235 (16.0)	<0.001	237 (11.9)	112 (10.0)	125 (14.4)	0.003
Substance use	292 (9.3)	127 (7.5)	165 (11.2)	<0.001	171 (8.6)	81 (7.2)	90 (10.4)	0.013
Type 1 diabetes	78 (2.5)	33 (2.0)	45 (3.1)	0.043	34 (1.7)	20 (1.8)	14 (1.6)	0.8
Type 2 diabetes	864 (27.4)	371 (21.9)	493 (33.7)	<0.001	439 (22.0)	208 (18.6)	231 (26.6)	<0.001
Prior PCI during the 6-month baseline^2^	73 (2.3)	40 (2.4)	33 (2.3)	0.8	47 (2.4)	28 (2.5)	19 (2.2)	0.7
Prior PCI during the entire time period before the index date	187 (5.9)	97 (5.7)	90 (6.2)	0.6	88 (4.4)	54 (4.8)	34 (3.9)	0.3
Prior CABG during the 6-month baseline^2^	15 (0.5)	6 (0.4)	9 (0.6)	0.3	7 (0.4)	4 (0.4)	3 (0.3)	>0.9
Prior CABG during the entire time period before the index date	34 (1.1)	14 (0.8)	20 (1.4)	0.14	14 (0.7)	8 (0.7)	6 (0.7)	>0.9
Prior MI during the 6-month baseline^6^	442 (14.0)	216 (12.7)	226 (15.4)	0.031	248 (12.4)	128 (11.4)	120 (13.8)	0.11
Prior MI during the entire time period before the index date	662 (21.0)	327 (19.4)	335 (22.9)	0.015	327 (16.4)	168 (15.0)	159 (18.3)	0.048
**hsCRP, mean (SD), median (IQR)**	2.37 (2.29), 1.50 (0.70, 3.10)	0.85 (0.47), 0.80 (0.40, 1.20)	4.13 (2.29), 3.40 (2.30, 5.60)	<0.001	2.25 (2.22), 1.40 (0.70, 3.00)	0.84 (0.47), 0.80 (0.40, 1.20)	4.08 (2.26), 3.40 (2.20, 5.50)	<0.001
**CV medication use, n (%)**								
Antihypertensives	1741 (55.3)	860 (50.9)	881 (60.3)	<0.001	1014 (51.0)	530 (47)	484 (55.8)	<0.001
Antihyperglycemics with CV benefits	0 (0.0)	0 (0.0)	0 (0.0)		0 (0.0)	0 (0.0)	0 (0.0)	
Statins	1254 (39.8)	666 (39.4)	588 (40.2)	0.7	708 (35.6)	407 (36.3)	301 (34.7)	0.4
Other lipid-lowering medications	319 (10.1)	143 (8.5)	176 (12.0)	<0.001	178 (9.0)	78 (7.0)	100 (11.5)	<0.001
Colchicine	31 (1.0)	18 (1.1)	13 (0.9)	0.6	17 (0.9)	9 (0.8)	8 (0.9)	0.8
Antiplatelet agents	405 (12.9)	185 (10.9)	220 (15.1)	<0.001	172 (8.7)	84 (7.5)	88 (10.1)	0.038
CVD medications	443 (14)	206 (12.2)	237 (16.2)	0.001	205 (10.3)	107 (9.6)	98 (113)	0.2
Thrombolytic agents	5 (0.2)	<3 (<0.5)	3 (0.2)	0.7	<3 (<0.5)	<3 (<0.5)	0 (0.0)	>0.9
Vasodilating agents	250 (7.9)	122 (7.2)	128 (8.8)	0.11	115 (5.8)	64 (5.7)	51 (5.9)	0.9
Anticoagulants	264 (8.4)	126 (7.5)	138 (9.4)	0.045	120 (6.0)	61 (5.4)	59 (6.8)	0.2
Other CVD medications	<3 (<0.1)	<3 (<0.1)	0 (0.0)	>0.9	0 (0.0)	0 (0.0)	0 (0.0)	
**Immunosuppressive medication, n (%)**								
Any immune suppressants	691 (21.9)	333 (19.7)	358 (24.5)	0.001	401 (20.1)	204 (18.2)	197 (22.7)	0.014
NSAIDs	651 (20.7)	322 (19.1)	329 (22.5)	0	385 (19.3)	198 (17.7)	187 (21.5)	0.031

1 Among the total sample (*N* = 3149), 1239 had STEMI (39.4%) and 1910 had NSTEMI (60.6%).

2 Cells in the table with values fewer than 3 patients were masked and or presented as "<3″ for compliance purposes related to patient identification.

3 Participants whose age group was unknown or missing (<0.5% of the sample) were included in the age group 55–64 years.

4 Comorbidities were identified by ICD-10-CM codes.

5 Selected auto-immune diseases included the following conditions (ICD-10-CM codes): rheumatoid arthritis (M05%, M06%), psoriasis (L40%), systemic lupus erythematosus (M32%), ankylosing spondylitis (M45%), ulcerative colitis (K51%), or Crohn's disease (K50%).

6 The baseline period was defined as 182 days before the index date, which was the first occurrence of AMI during the patient identification period. Abbreviations: AMI, acute myocardial infarction (type 1); CABG, coronary artery bypass grafting; CKD, chronic kidney disease; CV, cardiovascular; CVD, cardiovascular disease; FFS, fee-for-service; NSAIDs, non-steroidal anti-inflammatory drugs; PCI, percutaneous coronary intervention.

### 2.2 Revised 3-point MACE and individual MACE components

Patients with SI had a higher incidence rate of revised 3-point MACE (45.4 vs. 27.3 per 1000 PY) versus patients without SI (Table 2; Fig. 1A). In Cox regression models adjusted for patient baseline characteristics, SI was associated with significantly increased risk of revised 3-point MACE (HR: 1.39 [1.08–1.78]), corresponding to a 39% increase in risk during the follow-up period. Covariates associated with increased risk of revised 3-point were Medicaid payer group, hypertension, history of smoking, use of antiplatelet agents, and use of cardiovascular medications (all *p* < 0.05). Dyslipidemia was associated with lower risk of revised 3-point MACE (*p* < 0.05).

**Table 2:** Association of systemic inflammation with the occurrence of outcomes during follow-up (Table footnote^1^ is cited in Table body part).

1 Revised 3-point MACE, revised 4-point MACE, and individual MACE components were assessed in the full analytic sample (*N* = 3149). HF outcomes were assessed only among the subsample of patients without prior HF (*n* = 1988). HF outcomes were assessed as the time to the first HF event (whether a hospitalization or outpatient visit). HRs were obtained from the Cox proportional hazard models, controlling for patient demographics, clinical characteristics, and baseline medication use. Abbreviations: AMI, acute myocardial infarction (type 1); HF, heart failure; HR, hazard ratio; MACE, major adverse cardiovascular events; MI, myocardial infarction; SI, systemic inflammation; PY, person-years.

**Fig. 1A:**
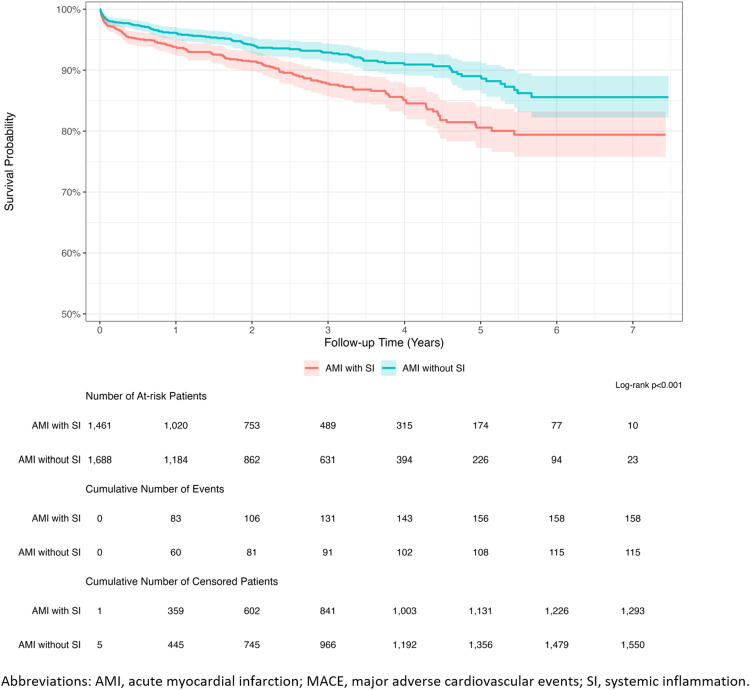
Kaplan-Meier curve for revised 3-point MACE. Abbreviations: AMI, acute myocardial infarction; MACE, major adverse cardiovascular events; SI, systemic inflammation.

Analysis of the individual components of revised 3-point MACE showed that patients with SI had a higher incidence rate of all-cause mortality (14.9 vs. 7.5 per 1000 PY), nonfatal MI (24.5 vs. 17.9 per 1000 PY), and nonfatal stroke (6.0 vs. 3.7 per 1000 PY) versus patients without SI (Table 2). In the Cox regression models, SI was associated with significantly increased risk of all-cause mortality (HR: 1.37 [1.07–1.75] (Fig. 1C). SI was not statistically associated with either nonfatal MI (HR: 1.16 [0.84–1.59]) or nonfatal stroke (HR: 1.42 [0.72–2.79]) (Table 2).

**Fig. 1C:**
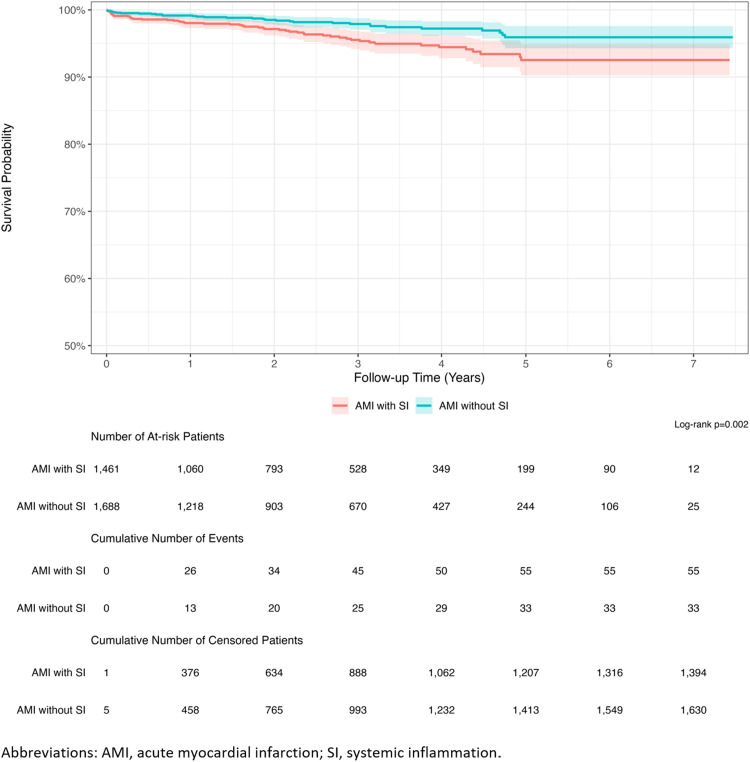
Kaplan-Meier curve for all-cause mortality. Abbreviations: AMI, acute myocardial infarction; SI, systemic inflammation.

In the subgroup of patients with NSTEMI-type index AMI (*n* = 1910; 60.6%), SI was associated with increased risk of revised 3-point MACE (*n* = 1910; HR: 1.36 [1.00–1.86], *p* = 0.049). No significant association was observed among the subgroup of patients with STEMI-type index AMI (*n* = 1239 [39.4%]; HR: 1.44 [0.96–2.18], *p* = 0.08).

### 2.3 Revised 4-point MACE and urgent revascularization

Patients with SI had a higher incidence rate of revised 4-point MACE (52.1 vs. 35.1 per 1000 PY) versus patients without SI (Table 2; Fig. 1B). In the Cox regression model adjusted for patient baseline characteristics, SI was associated with significantly increased risk of revised 4-point MACE (HR: 1.26 [1.01–1.58]). Covariates associated with increased risk of revised 4-point MACE were Medicaid payer group, hypertension, history of smoking, and use of antiplatelet agents (all *p* < 0.05). Dyslipidemia was associated with lower risk of revised 4-point MACE (*p* < 0.05).

**Fig. 1B:**
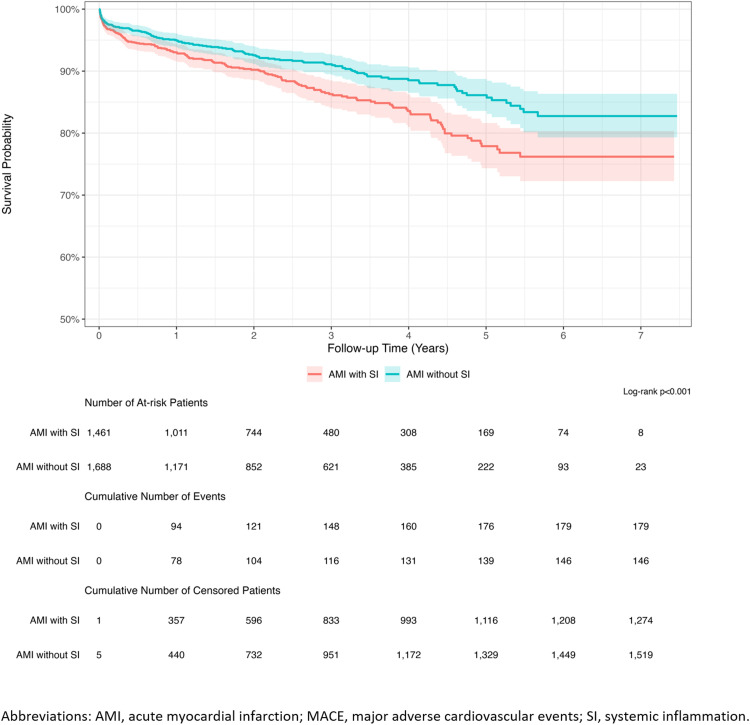
Kaplan-Meier curve for revised 4-point MACE. Abbreviations: AMI, acute myocardial infarction; MACE, major adverse cardiovascular events; SI, systemic inflammation.

Patients with SI had a slightly lower incidence of urgent revascularization (15.7 vs. 16.2 per 1000 PY) versus patients without SI. However, SI was not statistically associated with urgent revascularization (HR: 0.90 [0.63–1.30]) (Table 2) during the follow-up period.

### 2.4 Heart failure

Among the subsample of patients without prior HF (*n* = 1988), the incidence rate of an HF composite outcome, identified by either HF hospitalization or HF outpatient visit, was numerically higher among patients with versus without SI (72.8 vs. 51.6 per 1000 PY). SI was not statistically associated with the incidence of the HF composite (HR: 1.26 [0.99–1.60]; *p* = 0.06) (Table 2; Fig. 2A). Medicare Advantage payer group (OR, 1.95; *p* < 0.01) and greater comorbidity burden (QCI 1: OR, 1.71, *p* < 0.01; QCI 2+: OR, 1.68; *p* = 0.01) were associated with increased risk of the HF composite.

**Fig. 2A:**
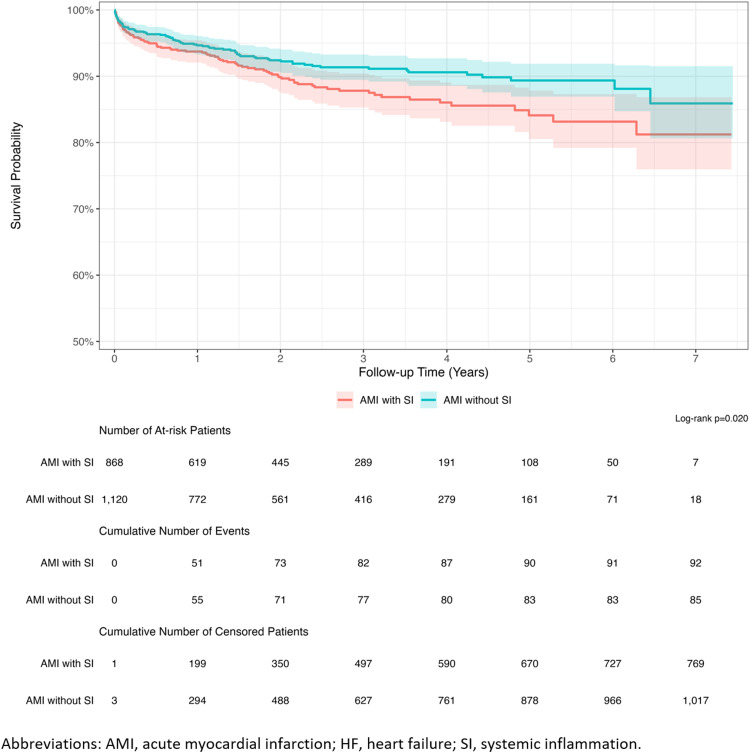
Kaplan-Meier curve for HF composite. Abbreviations: AMI, acute myocardial infarction; HF, heart failure; SI, systemic inflammation.

Patients with SI had a higher incidence rate of HF hospitalization (21.2 vs. 9.5 per 1000 PY) versus patients without SI (Table 2; Fig. 2B). SI was significantly associated with HF hospitalization (HR: 2.04 [1.26–3.30]; *p* < 0.01), corresponding to a 104% increase in the risk. Covariates associated with HF hospitalization were Medicare Advantage payer group, QCI score of 2+, and use of antiplatelet agents or CVD medications. The incidence rate of HF outpatient visits was higher among patients with SI (71.0 vs. 49.6 per 1000 PY), but SI was not statistically associated with increased risk of HF outpatient visits (HR: 1.26 [0.99–1.61]; *p* = 0.06) (Table 2; Fig. 2C).

**Fig. 2B:**
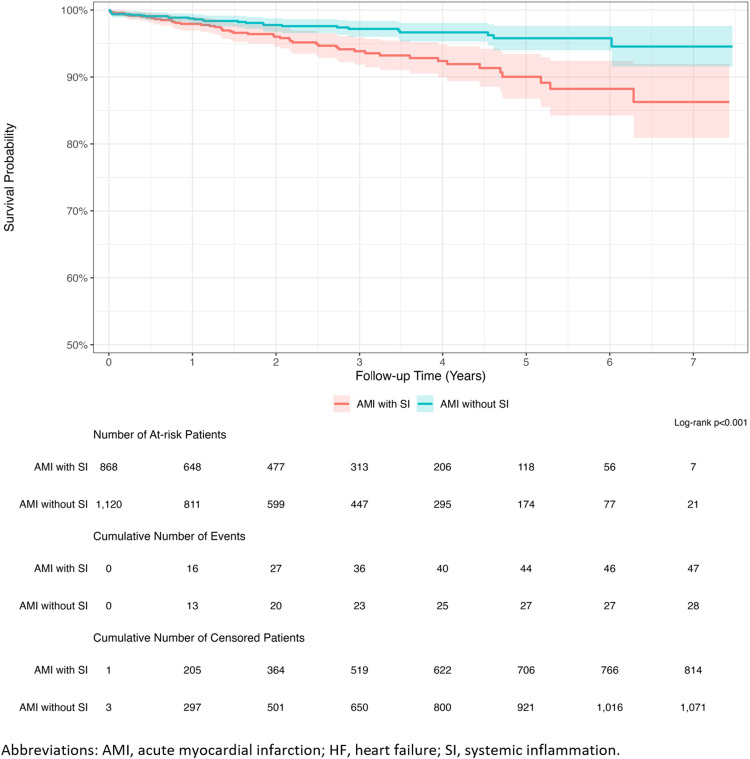
Kaplan-Meier curve for HF hospitalization. Abbreviations: AMI, acute myocardial infarction; HF, heart failure; SI, systemic inflammation.

**Fig. 2C:**
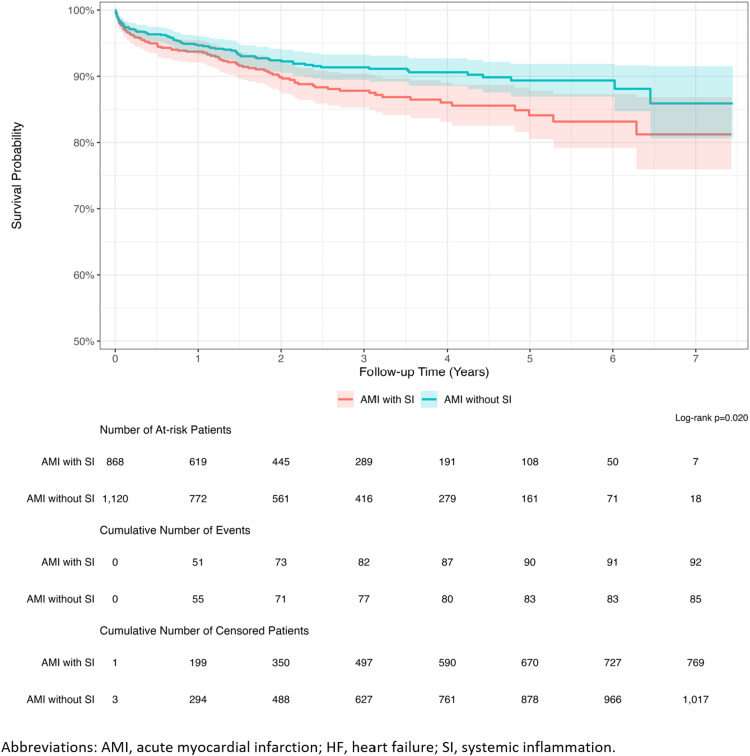
Kaplan-Meier curve for HF outpatient. Abbreviations: AMI, acute myocardial infarction; HF, heart failure; SI, systemic inflammation.

### 2.5 Study and source population

Among patients with AMI, the study and source populations were both characterized overall by a broad, heterogeneous mix of patients. The study sample (*N* = 3149) was similar to the source population (*N* = 715,962) in most demographic characteristics, including mean age (61 vs. 63 years), gender (69% vs. 63% male), race (60% vs. 58% White), and ethnicity (70% vs. 65% Non-Hispanic) (**Supplemental Table 1**). There were some variations between the study and source population in the distribution of patients by insurance type (e.g., 62% vs. 42% with commercial insurance, respectively) and geographic region. Regarding comorbidity burden, the study and source populations had similar proportions of patients with QCI scores of 1 (15% each) or 2 (10% vs. 12%, respectively), with larger variations in the distribution of patients with QCI scores of 0 (68% vs. 53%) or 3 (7% vs. 20%). Similar patterns were observed when stratifying the source population by hsCRP testing status (tested: *n* = 60,050; not tested: *n* = 655,912).

## 3 Discussion

This real-world, population-based cohort study was conducted to examine the association of SI, based on hsCRP, with cardiovascular events in a diverse population of patients with a history of AMI in the United States. SI was common among patients with a prior AMI, and patients who were female, over age 65 years, and who had more comorbidities were more likely to have SI at baseline. Over a median 3 years of follow-up, the presence of SI was associated with increased risk of all-cause mortality, revised 3-point MACE, revised 4-point MACE, and incident HF hospitalization. This study was conducted with a patient population that is demographically similar to the general population of patients with AMI in the United States [38]—average age in the 60–70-year range, a predominance of male patients, and comparable racial and ethnic distributions—and suggests that SI contributes to subsequent, unfavorable cardiovascular outcomes in patients who have experienced a type 1 AMI.

In our study, SI at baseline was associated with a significant increase in the risk of revised 3-point MACE, revised 4-point MACE, and all-cause mortality. Similar findings have been reported in other epidemiological studies despite variations in setting, study design, and population A retrospective cohort study of patients in Stockholm County, Sweden, who underwent hsCRP testing >30 days after MI, found that those with hsCRP levels ≥2 mg/L had approximately 1.28 times higher risk of MACE (nonfatal MI, nonfatal ischemic stroke, or cardiovascular death) and 1.42 times higher risk of death over a median follow-up period of 3.2 years [33]. Likewise, a retrospective analysis of 10,160 participants in the Atherosclerosis Risk in Communities (ARIC) cohort in the US revealed that participants with elevated hsCRP levels (≥3 mg/L over 2 study visits 6 years apart) had increased risk of coronary heart disease (CHD; HR: 1.51), ischemic stroke (HR: 1.70), and death (HR: 1.52) compared with patients with sustained low/moderate hsCRP over 15 years. The risk of these outcomes persisted in subanalyses with a 2 mg/L cutpoint (CHD: HR, 1.36; ischemic stroke: HR, 1.65; death: HR, 1.40) and in subanalyses excluding hsCRP levels >10 mg/L (CHD: HR, 1.46; ischemic stroke: HR, 1.59; death: HR, 1.42) [39]. Furthermore, a recent exploratory real-world study in the US indicated that patients with recent AMI and evidence of SI (determined by an hsCRP or CRP test value between 2 and 10 mg/L) had higher risk of mortality within 90 days after index discharge, compared with patients without SI (43.4% vs. 37.1%, respectively; *p* < 0.001) [40]. In our study, although SI at baseline (as indicated by ≥1 hsCRP test 2–10 mg/L) was not associated with a significant increase in the risk of nonfatal MI and nonfatal stroke, the risk for these outcomes was directionally positive. Differences in the magnitude of risks observed in our study and prior studies may reflect demographic or clinical heterogeneity (e.g., racial/ethnic and age distributions, the types of AMI included) as well as methodological heterogeneity (e.g., endpoint definitions, follow-up duration). Nonetheless, despite variations in settings, populations, and study designs, our findings and prior evidence consistently associate cardiovascular inflammation, defined by elevated hsCRP, with increased risk of adverse cardiovascular outcomes. Unlike prior studies conducted at single institutions or in regional databases outside the US, our study contributes additional evidence from a diverse population-based cohort representative of US clinical practice.

Building on these findings, subgroup analyses by AMI subtype in the present study revealed that SI was significantly associated with increased risk of revised 3-point MACE in patients with NSTEMI-type index AMI but not in patients with STEMI-type index AMI. Prior research assessing the relationship between hsCRP levels and prognosis among patients with STEMI has produced conflicting results, which have been attributed to between-study heterogeneity as well as inherent differences in pathophysiology between STEMI and NSTEMI [41,42]. Some studies show that patients with STEMI exhibit higher CRP levels than patients with NSTEMI [[43], [44], [45]], reflecting the abrupt plaque rupture, acute occlusion, and early inflammatory response that characterizes STEMI. However, patients with STEMI may conversely have lower overall long-term risk for recurrent events after the acute episode is managed than patients with NSTEMI [[46], [47], [48]]. Differences in pathophysiology, patient risk factors, and treatment strategies for STEMI versus NSTEMI [[48], [49], [50]], along with evidence of higher post-discharge cardiac and all-cause mortality for patients with NSTEMI [41,42], underscore the importance of characterizing the role of SI by type of AMI and subsequent cardiovascular outcomes. The potential for differences in inflammatory risk profiles by AMI subtype warrants further investigation to clarify these associations and their clinical implications.

We also assessed the association between SI and newly diagnosed HF after AMI and found a two-fold increased risk of HF hospitalization. Cardiovascular inflammation after AMI contributes to infarct size and ischemia-reperfusion damage, leading to left ventricular dysfunction and remodeling, which can contribute to the development of HF [10,51,52]. Prior observational studies have also shown that elevated hsCRP levels are associated with increased risk of HF. A single-center study of 4504 patients with a history of AMI in China found that patients with hsCRP levels in the third or fourth quartile had increased risks of in-hospital HF (OR: 1.36 and 1.41, respectively), postdischarge rehospitalization (HR: 1.33 and 1.80, respectively), and all-cause mortality (HR: 1.74 and 2.42, respectively), compared with patients with hsCRP levels in the first quartile [16]. The previously mentioned study of the ARIC cohort also found that hsCRP levels ≥2 mg/L and ≥3 mg/L were associated with increased risk of HF (HR: 1.37 and 1.60, respectively) over 15 years of follow-up [39]. Collectively, these findings indicate that elevated hsCRP levels may predict multiple adverse HF outcomes following an AMI, highlighting the need for ongoing strategies to mitigate these risks.

Therapeutic advances have reduced cardiovascular morbidity and mortality after AMI, largely through medical and surgical treatments (e.g., percutaneous coronary intervention and coronary artery bypass grafting) that have advanced the standard of care [[53], [54], [55], [56]]. Our cohort, comprising patients identified from 2016 to 2023 and followed through the end of 2023, reflects patients treated under the current standard of care for AMI and also shows that patients with SI are more likely than patients without SI to be treated with cardiovascular medications. Nonetheless, our findings indicate that unaddressed cardiovascular inflammation contributes to unmet clinical need in this patient population, indicating that current therapies may not fully control inflammation, which can persist for years after an AMI [57], contributing to unfavorable cardiovascular outcomes [53,55]. Awareness of the role of inflammation in cardiovascular outcomes is increasing. A 2025 American College of Cardiology Scientific Statement addressing inflammation and cardiovascular disease risk includes a call to action to screen patients for hsCRP, in combination with LDL-C, and provide anti-inflammatory interventions for patients with established ASCVD and evidence of residual inflammatory risk [58]. Elevated LDL-C has long been a target of clinical trials to reduce the risk of cardiovascular events [59,60]. However, observational studies have reported an inverse association between LDL-C and cardiovascular outcomes, known as the “lipid paradox” [[61], [62], [63]]. Our study is consistent with the literature, showing that patients with dyslipidemia at baseline had a lower risk of 3-point MACE.

The persistent risk associated with elevated hsCRP levels in our study suggests a need for anti-inflammatory therapies aimed at mitigating SI. Low-dose colchicine is currently the only approved medication to address cardiovascular inflammation, and it is intended to be used as an adjunct to lipid-lowering medications [64,65]. Two meta-analyses of randomized controlled trials of colchicine versus placebo showed that colchicine substantially reduces the risk of MACE and individual cardiovascular endpoints but can cause gastrointestinal events [66,67]. However, colchicine use may be limited by concerns about potential adverse effects at high doses and acute kidney injury, particularly in patients with CKD [68]. Additionally, the recently published Colchicine and Spironolactone in Patients with Myocardial Infarction/Synergy Stent Registry (CLEAR SYNERGY) trial produced mixed findings on the use of colchicine in clinical practice with patients who have experienced an AMI [69]. However, CLEAR SYNERGY was conducted during the COVID-19 pandemic, which impacted cardiovascular hospitalizations with potential underreporting of nonfatal cardiovascular events during that period [70]. Analyses of CLEAR SYNERGY data collected during the prepandemic period indicated a 22% reduction in the incidence of the primary endpoint, which was attenuated during the pandemic, with evidence of an interaction between pandemic phase and treatment effect [65]. In the Cholesterol Lowering via Bempedoic Acid, an ACL-Inhibiting Regimen Outcomes (CLEAR Outcomes) trial with patients with risk factors or history of cardiovascular events who were unable or unwilling to take statin therapy, elevated baseline hsCRP level (median measurement at trial entry: 2.30 mg/L) was associated with increased risk of MACE (HR: 1.43), cardiovascular death (HR: 2.00), and all-cause death (HR: 2.21) over 41 months [71] Randomized trials of new therapeutic monoclonal antibodies that target IL-6, such as tocilizumab, ziltivekimab, pacibekitug, and clazakizumab [32,[72], [73], [74], [75], [76]], are being conducted in diverse patient populations. Continued safety, efficacy, and effectiveness research of anti-inflammatory therapies is needed to optimize treatment for patients with unaddressed cardiovascular inflammation after AMI, potentially improving long-term cardiovascular outcomes in this population.

This study had some limitations. Our study was based on a sample of patients who had undergone hsCRP testing, which could impart the possibility of selection bias. The cause of death was not available, prohibiting the assessment of CV death. All-cause mortality data may be subject to underreporting, but there was no evidence to suggest that such potential underestimation was systematically different between the two cohorts. It is possible that the specific type and severity of MI were not accurately classified in the database. Left ventricular ejection fraction (LVEF) data are not available for HF phenotype. These limitations are inherent to the observational study design and the use of administrative claims data, which do not allow for the establishment of causality. In addition, the study period overlapped with the COVID-19 pandemic, which could have influenced hsCRP testing frequency, the prevalence of inflammation, and AMI presentation patterns. Future studies exploring potential effects of these pandemic-related factors on the association between SI and the risk of cardiovascular events could further enhance understanding of the impact of COVID-19 on cardiovascular health.

## 4 Conclusions

This large population-based study identified a substantially increased risk of revised 3-point MACE, revised 4-point MACE, incident HF hospitalization, and all-cause mortality over average follow-up period of 3 years among patients with type 1 AMI and SI. The findings suggest that hsCRP levels could be a prognostic marker of these outcomes among patients experiencing a type 1 AMI and who are treated under the current standard of care. The findings underscore substantial unmet clinical need in patients with type 1 AMI and highlight the importance of continuing to improve treatment for this patient population.

## Ethical review statement

This nonexperimental, retrospective, observational study was exempt from institutional review board review/approval. The research team accessed a limited dataset without individual identifiers, and only summary statistics were reported. This work complied with all relevant provisions of the US Health Insurance Portability and Accountability Act (HIPAA). Komodo Health approved the use of the dataset for this study.

## Prior presentation

Part of this work was presented at the Annual Society of Preventive Cardiology Congress, August 1–3, 2025, in Boston, MA.

## Data availability

All data supporting the conclusions of these analyses are presented in the manuscript or the supporting information. Details of additional data can be obtained from the corresponding author upon reasonable request. Komodo datasets are available for purchase from Komodo Health®.

## Funding

This work was funded by Novo Nordisk Inc., Plainsboro, NJ.

## CRediT authorship contribution statement

**Chi Nguyen:** Writing – review & editing, Writing – original draft, Supervision, Investigation, Conceptualization. **Amanda M. Ackermann:** Writing – review & editing, Writing – original draft, Investigation, Conceptualization. **Erica Marieb:** Writing – review & editing, Writing – original draft, Methodology, Investigation, Conceptualization. **Jeffrey R. Skaar:** Writing – review & editing, Writing – original draft, Methodology, Investigation, Conceptualization. **Wing Chow:** Writing – review & editing, Writing – original draft, Investigation, Conceptualization. **Radha Ryali:** Writing – review & editing, Writing – original draft, Methodology, Investigation, Conceptualization. **Lyuba Popadic:** Visualization, Formal analysis, Data curation. **Xiyuan Wu:** Visualization, Formal analysis, Data curation. **Xinshuo Ma:** Visualization, Formal analysis, Data curation. **Kathleen E․ Kearney:** Writing – review & editing, Writing – original draft.

## Declaration of competing interest

The authors declare the following financial interests/personal relationships which may be considered as potential competing interests:

Xiyuan Wu reports financial support was provided by Novo Nordisk Inc. If there are other authors, they declare that they have no known competing financial interests or personal relationships that could have appeared to influence the work reported in this paper.

Xinshuo Ma reports financial support was provided by Novo Nordisk Inc. If there are other authors, they declare that they have no known competing financial interests or personal relationships that could have appeared to influence the work reported in this paper.

Wing Chow reports a relationship with Novo Nordisk Inc that includes: employment and equity or stocks. If there are other authors, they declare that they have no known competing financial interests or personal relationships that could have appeared to influence the work reported in this paper.

Radha Ryali reports financial support was provided by Novo Nordisk Inc. Radha madhavi Ryali reports a relationship with Novo Nordisk Inc that includes: employment. Co-author was employed by the company during when the study was conducted If there are other authors, they declare that they have no known competing financial interests or personal relationships that could have appeared to influence the work reported in this paper.

Lyuba Popadic reports financial support was provided by Novo Nordisk Inc. If there are other authors, they declare that they have no known competing financial interests or personal relationships that could have appeared to influence the work reported in this paper.

Kathleen Kearney reports a relationship with Novo Nordisk Inc that includes: consulting or advisory. Kathleen Kearney reports a relationship with Abbott that includes: consulting or advisory and speaking and lecture fees. Kathleen Kearney reports a relationship with Boston Scientific (Institutional) that includes: consulting or advisory and speaking and lecture fees. Kathleen Kearney reports a relationship with Medtronic Inc that includes: consulting or advisory and speaking and lecture fees. Kathleen Kearney reports a relationship with Asahi that includes: consulting or advisory and speaking and lecture fees. Kathleen Kearney reports a relationship with Teleflex that includes: consulting or advisory and speaking and lecture fees. Kathleen Kearney reports a relationship with Johnson and Johnson Medtech (Shockwave and Abiomed previously) that includes: consulting or advisory and speaking and lecture fees. Kathleen Kearney reports a relationship with Fastwave (an intravascular lithotripsy company, I have not executed these but have the ability to do so) that includes: equity or stocks. If there are other authors, they declare that they have no known competing financial interests or personal relationships that could have appeared to influence the work reported in this paper.

Jeffrey Skaar reports a relationship with Novo Nordisk Inc that includes: employment and equity or stocks. Jeffrey Skaar reports a relationship with Trinity Life Sciences that includes: equity or stocks. If there are other authors, they declare that they have no known competing financial interests or personal relationships that could have appeared to influence the work reported in this paper.

Erica Marieb reports a relationship with Novo Nordisk Inc that includes: employment and equity or stocks. If there are other authors, they declare that they have no known competing financial interests or personal relationships that could have appeared to influence the work reported in this paper.

Chi Nguyen reports a relationship with Novo Nordisk Inc that includes: employment and equity or stocks. If there are other authors, they declare that they have no known competing financial interests or personal relationships that could have appeared to influence the work reported in this paper.

Amanda Ackermann reports a relationship with Novo Nordisk Inc that includes: employment and equity or stocks. If there are other authors, they declare that they have no known competing financial interests or personal relationships that could have appeared to influence the work reported in this paper.

## Appendix Supplementary materials

Image, application 1
