# Supplementary material for: Association of systemic inflammation with major adverse cardiovascular events in patients with acute myocardial infarction
Source: Am J Prev Cardiol. 2026 Mar 24;29:101574. doi: 10.1016/j.ajpc.2026.101574 (PMC13329531; doi:10.1016/j.ajpc.2026.101574)
Supplement: Supplementary file 1 [file mmc1.docx]

**SUPPLEMENTARY MATERIAL**

**Manuscript Title:**

Association of Systemic Inflammation with Major Adverse Cardiovascular Events in Patients with Acute Myocardial Infarction

**Short Title:**

Association of Systemic Inflammation with MACE in Patients with AMI

**Authors:**

Chi Nguyen^1^, PhD; Amanda M. Ackermann^1^, MD, PhD; Erica Marieb^1^, PhD; Jeffrey R. Skaar^1^, PhD; Wing Chow^1^, PharmD, MPH; Radha Ryali^1^, MS; Lyuba Popadic^2^; Xiyuan Wu^2^, MS; Xinshuo Ma^2^; Kathleen Kearny, MD^3^

**Affiliations:**

^1^Novo Nordisk Inc., Plainsboro, NJ, USA

^2^Komodo Health, New York, NY, USA

^3^University of Washington School of Medicine, Seattle, WA, USA

**Corresponding Author:**

Chi Nguyen, PhD

Novo Nordisk Inc.

800 Scudders Mill Rd.

Plainsboro, NJ 08536, USA

**Supplemental Table 1. List of cardiovascular, immunosuppressive, and non-steroidal anti-inflammatory medications identified from medical and pharmacy claims during the baseline period.**

| **Cardiovascular medications** |
| --- |
| anti-hypertensives (diuretics, beta blocker, calcium channel blockers, angiotensin converting enzyme inhibitors,  angiotensin II receptor blocker, combination formulations, and others) |
| anti-arrhythmic agents |
| anti-coagulants |
| anti-platelet agents (cyclooxygenase inhibitors, adenosine diphosphate receptor antagonists,  glycoprotein IIb/IIA inhibitors, phosphodiesterase inhibitors, P2Y12 receptor blockers, and others) |
| anti-hyperglycemics with CV benefits (glucagon-like peptide-1 agonists,  sodium-glucose cotransporter-2 inhibitors) |
| statins (high vs. low intensity statins) |
| other lipid-lowering medications (PCSK9 inhibitors, fibrates, ezetimibe, bile acid sequestrants,  and combination formulations, and others) |
| colchicine |
| other CVD medications |

| **Immunosuppressive medications** |
| --- |
| **General immune** |
| steroids (i.e., prednisone, methylprednisolone, dexamethasone, budesonide, triamcinolone) |
| methotrexate |
| azathioprine |
| hydroxychloroquine |
| sulfasalazine |
| mycophenolate mofetil |
| dapsone (mostly used in dermatology-related autoimmune conditions) |
| **Innate immunity** |
| anti-IL-1 (i.e., anakinra, canakinumab, rilonacept) |
| anti-IL-6 (i.e., tocilizumab, sarilumab) |
| anti-TNF (i.e., etanercept, infliximab, adalimumab, certolizumab pegol, golimumab) |
| anti-integrin (i.e., natalizumab, vedolizumab) |
| **Adaptive immunity – B cells** |
| anti-CD20 (i.e., rituximab, ibritumomab, obinutuzumab, ocrelizumab, ofatumumab) |
| B-cell growth factor targeting (i.e., belimumab) |
| adaptive immunity–T cells (i.e., cyclosporine, everolimus leflunomide, sirolimus, tacrolimus) |
| T-cell co-stimulation and activation (i.e., abatacept) |
| **Adaptive immunity – cytokines** |
| anti-IL-17 (i.e., secukinumab, ixekizumab, brodalumab) |
| anti-IL-23 (i.e., guselkumab, risankizumab, tildrakizumab) |
| anti-IL-12/23 (i.e., ustekinumab) |
| anti-IL-5 (i.e., mepolizumab, reslizumab, benralizumab) |
| anti-IL-4/13 (i.e., dupilumab, tralokinumab-ldrm) |
| IgE targeting (i.e., omalizumab) |
| **Small molecule targeting medications** |
| JAK inhibitors (i.e., abrocitinib, baricitinib, deucravacitinib, ruxolitinib, tofacitinib, upadacitinib) |
| **Sphingosine-1-phosphate receptor modulator (S1P modulators)** |
| fingolimod, siponimod, ozanimod, ponesimod |
| **Drugs used in multiple sclerosis** |
| interferon beta, glatiramer acetate, teriflunomide, dimethyl fumarate, diroximel fumarate,  monomethly fumarate, cladribine, natalizumab, alemtuzumab |

| **Non-steroidal anti-inflammatory drugs (NSAIDs)** |
| --- |
| diclofenac |
| diflunisal |
| etodolac |
| fenoprofen |
| flurbiprofen |
| ibuprofen |
| indomethacin |
| ketoprofen |
| ketorolac |
| mefenamic acid |
| meloxicam |
| nabumetone |
| naproxen |
| oxaprozin |
| piroxicam |
| sulindac |
| tolmetin |
| celecoxib |
| aspirin |

Abbreviations: CV, cardiovascular; CVD, cardiovascular disease; IgE, immunoglobulin E; IL, interleukin; JAK, Janus kinase; PCSK9, proprotein convertase subtilisin/kexin-type 9; TNF, tumor necrosis factor.

**Supplemental Table 2. Study and source population.**

|  | **Study population** | **Source population^1^** | | |
| --- | --- | --- | --- | --- |
|  | **All eligible patients with AMI** | **All adult patients with AMI** | **Patients with AMI and tested for hsCRP** | **Patients with AMI and not tested for hsCRP** |
| **Number of patients, n (%)** | 3,149 (100) | 715,962 (100) | 60,050 (100) | 655,912 (100) |
| **Age on index date, mean (SD)** | 61 (11) | 63 (13) | 61 (12) | 63 (13) |
| **Age group, n (%)** |  |  |  |  |
| 18–44 | 223 (7) | 48,760 (7) | 4,457 (7) | 44,303 (7) |
| 45–54 | 634 (20) | 128,131 (18) | 11,928 (20) | 116,203 (18) |
| 55–64 | 1,302 (41) | 246,624 (34) | 22,480 (37) | 224,144 (34) |
| 65–74 | 588 (19) | 143,455 (20) | 11,556 (19) | 131,899 (20) |
| 75+ | 402 (13) | 148,992 (21) | 9,629 (16) | 139,363 (21) |
| **Gender, n (%)** |  |  |  |  |
| Male | 2,184 (69) | 454,258 (63) | 37,437 (62) | 416,821 (64) |
| Female | 932 (30) | 255,304 (36) | 21,982 (37) | 233,322 (36) |
| Unknown/missing | 33 (1) | 6,400 (1) | 631 (1.1) | 5,769 (1) |
| **Race, n (%)** |  |  |  |  |
| White/Caucasian | 1,879 (60) | 412,107 (58) | 33,739 (56) | 378,368 (58) |
| Black/African American | 242 (8) | 90,452 (13) | 7,739 (13) | 82,713 (13) |
| American Indian, Alaska Native, or Asian | 197 (6) | 29,272 (4) | 2,910 (5) | 26,362 (4) |
| Other | 245 (8) | 39,179 (6) | 4,106 (7) | 35,073 (5) |
| Unknown/missing | 586 (19) | 144,952 (20) | 11,556 (19) | 133,396 (20) |
| **Ethnicity, n (%)** |  |  |  |  |
| Hispanic | 410 (13) | 87,234 (12) | 9,494 (16) | 77,740 (12) |
| Non-Hispanic | 2,195 (70) | 463,748 (65) | 39,422 (66) | 424,326 (65) |
| Unknown/missing | 544 (17) | 164,980 (23) | 11,134 (19) | 153,846 (23) |
| **Insurance type, n (%)** |  |  |  |  |
| Commercial health insurance | 1,948 (62) | 298,448 (42) | 27,091 (45) | 271,357 (41) |
| Medicare (Advantage and FFS) | 871 (28) | 279,925 (39) | 20,602 (34) | 259,323 (40) |
| Medicaid | 330 (10) | 134,281 (19) | 12,215 (20) | 122,066 (19) |
| Other/unknown | 0 (0) | 3,308 (0.5) | 142 (0.2) | 3,166 (0.5) |
| **Geographic region, n (%)** |  |  |  |  |
| Northeast | 687 (22) | 166,943 (23) | 15,506 (26) | 151,437 (23) |
| Midwest | 379 (12) | 173,487 (24) | 10,888 (18) | 162,599 (25) |
| South | 1,244 (40) | 244,466 (34) | 18,764 (31) | 225,702 (34) |
| West | 839 (27) | 123,988 (17) | 14,503 (24) | 109,485 (17) |
| Other/unknown | 0 (0) | 7,078 (1) | 389 (1) | 6,689 (1) |
| **Quan-Charlson Comorbidity Index (QCI)** |  |  |  |  |
| Mean (SD) | 0.62 (1.13) | 1.29 (1.91) | 1.45 (1.99) | 1.28 (1.90) |
| 0, n (%) | 2,143 (68) | 380,387 (53) | 29,402 (49) | 350,985 (54) |
| 1, n (%) | 469 (15) | 105,194 (15) | 9,257 (15) | 95,937 (15) |
| 2, n (%) | 308 (10) | 85,943 (12) | 7,712 (13) | 78,231 (12) |
| 3+, n (%) | 229 (7) | 144,438 (20) | 13,679 (23) | 130,759 (20) |

^1^Continuous enrollment during 6 months before the index date.

Abbreviations: AMI, acute myocardial infarction; FFS, fee-for-service; hsCRP, high-sensitivity C-reactive protein; SD, standard deviation.

**Supplemental Figure 1. Study sample selection flow diagram.**

Patients ≥18 years admitted with type 1 AMI (index date) during identification period

**(N = 2,053,246)**

Patients with an eligible hsCRP test within ±1 year of the index date

**(N = 10,355)**

No diagnosis for major cancer, ESRD, CABG, severe hepatic disease (hepatic encephalopathy, ascites, or hepatic cirrhosis), or

evidence of chronic infectious diseases (hepatitis, HIV, tuberculosis)

**(N = 7,813)**

Continuous health plan enrollment for ≥6 months before

and ≥1 day after the index date

**(Final study sample; N = 3,149)**

**No**

**systemic inflammation**

**(n = 1,688)**

**Systemic inflammation**

**(n = 1,461)**

Abbreviations: AMI, acute myocardial infarction (type 1); CABG, coronary artery bypass grafting; ESRD, end-stage renal disease; hsCRP, high-sensitivity C-reactive protein.
